# Chronic prurigo in an adolescent patient successfully controlled with dupilumab

**DOI:** 10.1093/skinhd/vzag050

**Published:** 2026-04-29

**Authors:** Mohammed Nasser Al-Abdulla, Noof Al-Qahtani, Shahd M Younis, Syed Rizvi, Amro Elfaieg, Joerg Buddenkotte, Martin Steinhoff

**Affiliations:** Department of Dermatology and Venereology, Hamad Medical Corporation, Doha, Qatar; Dermatology Institute, Hamad Medical Corporation, Doha, Qatar; Translational Research Institute, Academic Health System, Hamad Medical Corporation, Doha, Qatar; Weill Cornell Medicine-Qatar, School of Medicine, Doha, Qatar; Department of Dermatology and Venereology, Hamad Medical Corporation, Doha, Qatar; College of Medicine, Qatar University,Doha, Qatar; Translational Research Institute, Academic Health System, Hamad Medical Corporation, Doha, Qatar; Department of Laboratory Medicine and Pathology, Hamad Medical Corporation, Doha, Qatar; Department of Laboratory Medicine and Pathology, Hamad Medical Corporation, Doha, Qatar; Department of Dermatology and Venereology, Hamad Medical Corporation, Doha, Qatar; Dermatology Institute, Hamad Medical Corporation, Doha, Qatar; Translational Research Institute, Academic Health System, Hamad Medical Corporation, Doha, Qatar; Department of Dermatology and Venereology, Hamad Medical Corporation, Doha, Qatar; Dermatology Institute, Hamad Medical Corporation, Doha, Qatar; Translational Research Institute, Academic Health System, Hamad Medical Corporation, Doha, Qatar; Weill Cornell Medicine-Qatar, School of Medicine, Doha, Qatar; College of Medicine, Qatar University,Doha, Qatar; Department of Dermatology, Weill Cornell Medicine, New York, NY, USA; School of Life Sciences, Hamad-bin-Khalifa University, Doha, Qatar

## Abstract

Chronic prurigo, formerly termed prurigo nodularis, is a chronic skin condition characterized by intensely itchy nodules and papules. It primarily affects adults, particularly middle-aged women. The aetiology of chronic prurigo is unclear, but it can be associated with primary skin dermatoses and systemic conditions. Minimal data on chronic prurigo in children are available in the literature. While systemic therapies for chronic prurigo have significant side effects, targeted biologic and small-­molecule inhibitors offer a promising treatment option with a good benefit–risk ratio. We report one of the first cases of an adolescent with chronic prurigo that was successfully controlled with dupilumab. Dupilumab treatment resulted in a fast, significant and sustainable improvement in the signs and symptoms of chronic prurigo, with favourable safety and tolerability profiles, highlighting its potential for recalcitrant cases and long-term remission.

## Teaser

Chronic prurigo, formerly termed prurigo nodularis, is a chronic skin condition characterized by intensely itchy nodules and papules that primarily affects adults, particularly middle-aged women. The aetiology is unclear, but it can be associated with primary skin dermatoses and systemic conditions. Minimal data are available in the literature for paediatric patients. While systemic therapies for chronic prurigo have significant side effects, targeted biologic and small-molecule inhibitors offer a promising treatment option with a good benefit–risk ratio. We report an adolescent with chronic prurigo that was successfully controlled with dupilumab. Dupilumab resulted in a fast, significant and sustainable improvement in the signs and symptoms of chronic prurigo, with a favourable safety and tolerability profile.

What is already known about this topic?

- Chronic prurigo is characterized by intensely itchy, nodules and papules, often with central excoriation.
- The management of chronic prurigo is challenging, especially in children.
- Topical treatments are often ineffective and systemic therapies have significant side effects or are contraindicated in children and adolescents.

What does this study add?

- Treatment of an adolescent with chronic prurigo with an anti-interleukin-4 receptor alpha antibody (dupilumab) resulted in a fast, significant and sustainable improvement, with a favourable safety profile.

Chronic prurigo, formerly known as prurigo nodularis, is a chronic inflammatory skin condition characterized by intensely pruritic, symmetric, hyperkeratotic nodules and papules, often with central excoriation, that leads to the induction of an itch–scratch cycle, negatively affecting patients’ quality of life. The disease manifests as numerous symmetrically distributed nodules on the extensor aspects of the extremities. The central upper back is usually spared as it is difficult to reach, creating a butterfly sign. Chronic prurigo primarily affects adults, particularly middle-aged women. The mean age at onset is reported to be 50.9 years.^1^ Paediatric chronic prurigo is rare, affecting 21.6 per 100 000 children and adolescents.^2^ Notably, chronic prurigo is a distinct disease entity, as recently highlighted by a European task force, and is not necessarily secondary to skin disorders like atopic dermatitis (AD) or systemic associations like chronic kidney disease.^3,4^

The pathogenesis of chronic prurigo is highlighted by elevated expresssion of interleukin (IL)-4Rα expression in lesions. Additionally, IL-4Rα is found on fibroblasts, and is implicated in IL-4- and IL-13-mediated cutaneous fibrosis, an important mechanism in the pathogenesis of chronic prurigo.^5^ Treatment with dupilumab blocks this pathway, potentially breaking the pathologic itch–scratch cycle. Dupilumab is approved for patients aged ≥6 months with AD and for chronic prurigo in adults aged ≥18 years. There are only sparse reports about the effects and adverse events of dupilumab in childhood chronic prurigo. Treatment options for chronic prurigo in paediatric and adolescent patients are limited compared with those for adults, and may have an altered adverse event profile, indicating the need for safe and effective approved medications.

We present one of the first reports of the successful control of chronic prurigo in an adolescent patient, demonstrating a complete and sustained response and rapid improvement in Numeric Rating Scale-11 (NRS11) itch score (from 8/10 at baseline to 0/10 after 6 months of dupilumab treatment). There was sustainable control of the signs and symptoms of chronic prurigo in the patient and further tapering of dupilumab over 2 years.

## Case report

**Figure 1:**
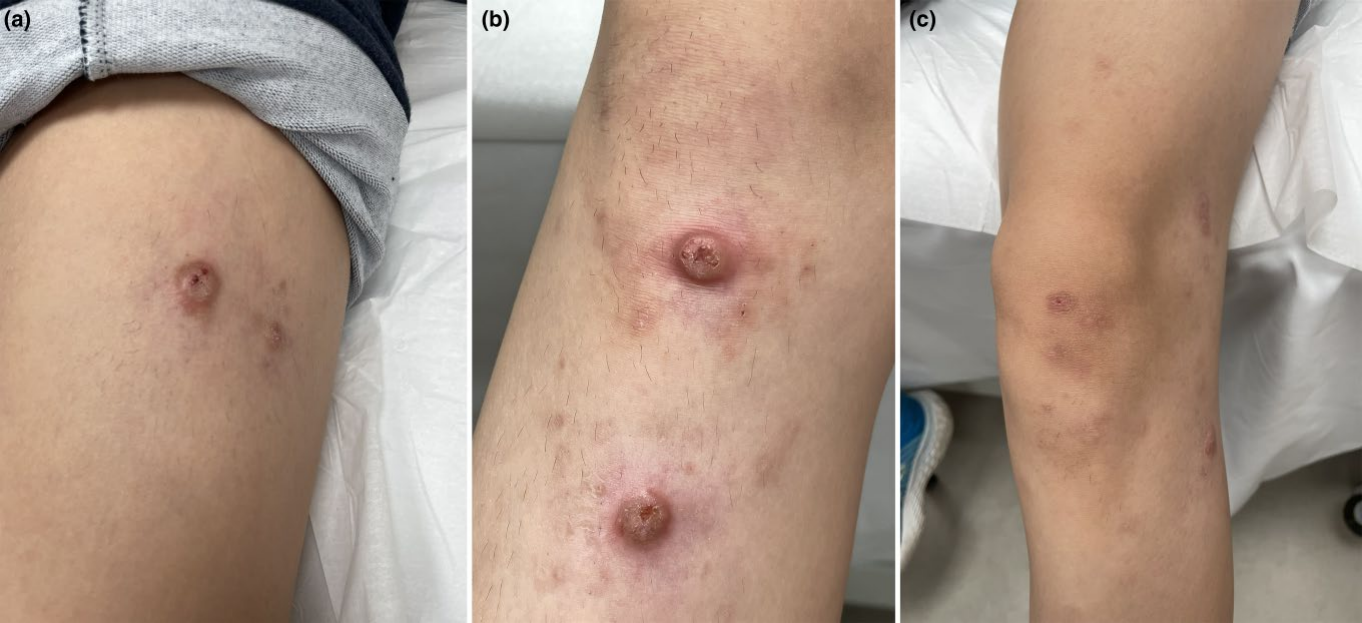
A 13-year-old Northeast Asian girl with chronic prurigo presented with multiple nodules, which were partly umbillicated and erosive, with dark-yellow crustations, on a background of erythema and surrounding excoriation marks on her (a) thighs and (b, c) lower legs.

A 13-year-old Northeast Asian girl presented with multiple nodules with dark-yellow crustations on her thighs and lower legs of 1 year’s duration (Figure 1). She had a history of well-controlled childhood AD and had been off treatment since the age of 6–7 years. The patient and her mother noted that the current lesions were different in nature from her childhood AD. The patient’s grandfather had AD. Medium-potency topical steroids had been prescribed for the current nodules by private clinics, but they did not respond to treatment. At the initial visit to our clinic, the patient presented with an NRS11 itch score of 8/10, a SCORing Atopic Dermatitis (SCORAD) of 21.5 and a Children’s Dermatology Life Quality Index (CDLQI) of 15/30; the condition was affecting her sleep and she had a total IgE level of 181.00 kunits L^–1^.

**Figure 2:**
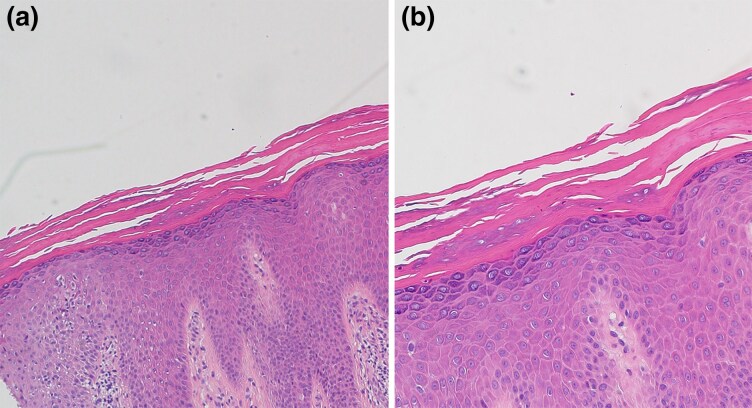
(a) Skin punch biopsy showing the histological features of chronic prurigo, including elongated rete ridges, acanthosis hypergranulosis, hyperkeratosis and mild dermal mixed inflammatory infiltrate (haematoxylin and eosin stain, magnification ×10). (b) Hypergranulosis, hyperkeratosis and parakeratosis are more apparent on higher magnification (haematoxylin and eosin stain, magnification ×20).

A 4-mm punch biopsy was taken from the patient’s left anterior thigh to confirm the clinical diagnosis of chronic prurigo. Clobetasol was used for 10 days under occlusion with plastic wrap as a bridging treatment while the histology results were pending. Histopathological assessment revealed hyperkeratosis, parakeratosis, hypergranulosis and acanthosis in the epidermis, with perivascular and periadnexal lymphohistiocytic infiltrate in the dermis. No neutrophils, eosinophils or fungi were found. The subcutis was normal. The absence of neutrophils/eosinophils indicated a chronic, noninfectious, nonallergic process, supporting the diagnosis of chronic prurigo (Figure 2).

**Figure 3:**
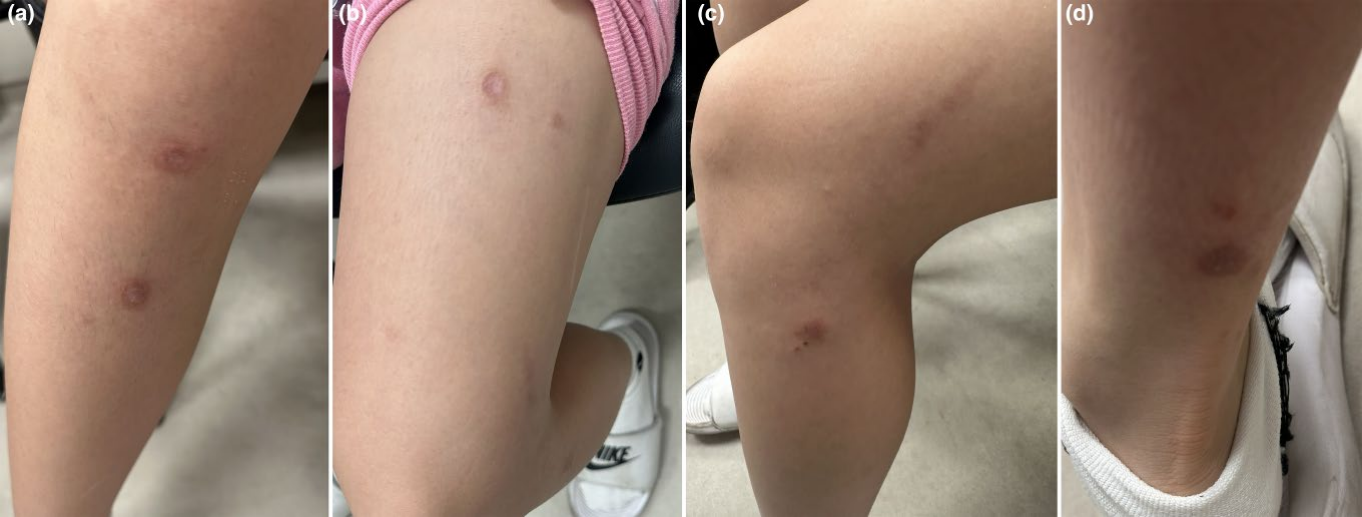
The same patient presenting with mildly elevated and hyperpigmented papules and nodules on her (a, b) thighs and (c, d) lower legs 6 months after monotherapy with dupilumab 300 mg every 2 weeks.

**Figure 4:**
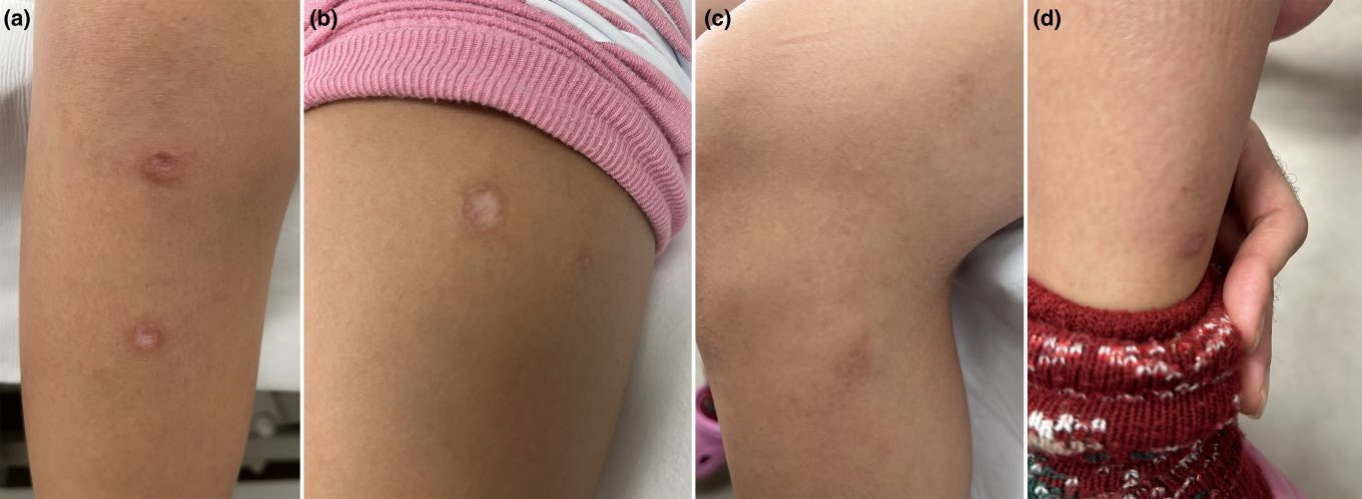
The same patient presenting with almost completely flat and asymptomatic papules and plaques with mild remnant dyspigmentation on her (a, b) thighs and (c, d) lower legs 12 months after monotherapy with dupilumab 300 mg every 2 weeks.

Following histopathological confirmation and discussion with the family regarding the safety and efficacy of treatment options [conventional immunosuppressants vs. biologics and Janus kinase (JAK) inhibitors], dupilumab monotherapy was preferred and started at 600 mg at week 0, followed by 300 mg biweekly. Within 1 month of dupilumab treatment, pruritus improved (1-month NRS11 score: 5/10; 4-month NRS11 score: 2/10). After 6 months, an NRS11 score of 0/10 was achieved. At this time, the patient’s lesions were asymptomatic, mildly raised and hyperpigmented (Figure 3). At 1 year of treatment, when the lesions had nearly completely flattened and were asymptomatic, with only mild residual dyspigmentation, the dosage of dupilumab was reduced to 300 mg every 4 weeks (Figure 4). The patient was followed up regularly and the treatment regimen was tapered over the next 2 years to 300 mg every 8 weeks without any relapses or side effects, and a sustainable NRS11 score of 0/10.

## Discussion

Chronic prurigo is a skin disorder in its own right with clinical findings of chronic intensely pruritic symmetric hyperkeratotic nodules and papules, often with central excoriation. Chronic prurigo mainly affects adults, particularly middle-aged women; paediatric cases are less common. Topical anti-inflammatory agents like glucocorticosteroids and calcineurin inhibitors are often ineffective and require long-term use. Systemic therapies have significant potential side effects (e.g. ciclosporin A and methotrexate) or are contraindicated (e.g. thalidomide). Yet, biologics such as dupilumab and nemolizumab or JAK inhibitors have been approved for the treatment of childhood or adolescent chronic prurigo. Type 2 inflammatory processes are crucial in paediatric chronic prurigo, despite the poorly studied neuroimmunology compared with adults. The immunological overlap with AD and the impact of cytokines such as IL-4, IL-13 or IL-31 on regulating pruritus are key to its pathogenesis.^6^ In the context of chronic prurigo, IL-4 and IL-13 also stimulate fibroblast proliferation, migration and the production of profibrotic factors such as transforming growth factor-β, collagen and periostin. This process contributes to exaggerated tissue repair and the characteristic skin fibrosis seen in patients with chronic prurigo, with some chronic lesions demonstrating elevated levels of IL-17. This neuroimmune shift highlights the importance of targeted biologic and small-molecule inhibitors that offer a good benefit–risk ratio in providing a safe, long-term treatment option with long-lasting remission.^7^

A PubMed search using the terms ((dupilumab) AND (prurigo nodularis)) AND (pediatric), retrieved 10 publications. Our findings are in accordance with other case reports that showcased improved pruritus starting at week 2 of treatment with peak improvement by week 12 and resolution of skin lesions by 3 months.^8,9^ The case of an 11-year-old child who did not respond to anti-JAK1 systemic therapy but responded well to dupilumab emphasizes the need for precision medicine in clinics to identify the most effective therapeutic intervention.^10^ Here, we report one of the first documented cases of chronic prurigo in an adolescent that was successfully controlled with dupilumab with sustained effect after tapering. Rapidly acting, sustainable efficacy and excellent safety agents like dupilumab appear to be a suitable treatment option for recalcitrant chronic prurigo in children and adolescents. Placebo-controlled randomized clinical trials or large cohort studies are needed to confirm the benefit of dupilumab in these patient cohorts.
